# Dr. Roberts Bartholow

**Published:** 1904-06

**Authors:** 


Dr. Roberts Bartholow, of Philadelphia, died at his home May 10, 1904, aged 72 years. He was a distinguished author and teacher, first at Cincinnati, and then at Philadelphia, and had been an active and honorary member of numerous medical societies.

